# Foundations for growth: lived experiences of nurses one year after a clinical nursing introduction programme

**DOI:** 10.1186/s12912-026-05210-w

**Published:** 2026-08-11

**Authors:** Fredrika Sundberg, Mia Berglund, Anna Kjellsdotter

**Affiliations:** 1 Research and Development Centre, Skaraborg Hospital Skövde, Region Västra Götaland, Lövängsvägen 1, Skövde, 54949 Sweden; 2 School of Health Sciences, University of Skövde, Skövde, Sweden

**Keywords:** Nurse transition, Clinical nursing introduction programme, Professional identity, Caring, Self-awareness, Reflective practice, Qualitative research, Descriptive phenomenology, Continuous professional development (CPD)

## Abstract

**Background:**

Discrepancies between academic nursing education and the realities of clinical practice may contribute to challenges during the transition from student to professional nurse. To address this, many countries and hospitals have implemented clinical introduction programmes for newly graduated nurses. However, limited knowledge exists regarding how these programmes influence nurses’ experiences of transitioning into their professional role over time.

**Objectives:**

This study seeks to explore the lived experiences of nurses one year after completing a clinical nursing introduction programme, two years post-graduation.

**Design:**

The study adopted a qualitative descriptive phenomenological design.

**Setting:**

The setting was a general hospital in southwest Sweden.

**Participants:**

The participants comprised 12 registered nurses two years after graduation who had completed a one-year clinical nursing introduction programme.

**Methods:**

Semi-structured interviews were conducted and analysed using qualitative thematic analysis grounded in descriptive phenomenology.

**Findings:**

Three themes were identified: A hunger for more, responsibility for being a caring nurse, and becoming self-aware and self-confident. Two years post-graduation and one year after completing the clinical nursing introduction programme, nurses expressed a strong drive for ongoing professional and personal growth, characterized by curiosity and a desire for autonomy. They emphasized responsibility for delivering patient-centred care, often prioritizing caring values to the extent of considering leaving workplaces that failed to support these ideals. Finally, participants reported increased confidence and self-awareness, viewing help-seeking as a sign of professional security rather than weakness. This development was supported by collegial relationships and reflective discussions on ethical dilemmas.

**Conclusions:**

The clinical nursing introduction programme may provide an important foundation for ongoing professional and personal growth. The findings suggest that protected time, peer interaction, reflective practice, and supportive management were perceived as contributing to a developmental trajectory from ‘being’ to ‘becoming’ a nurse, while supporting lifelong learning, caring values, and clinical confidence. However, these perceived longer-term benefits should be interpreted with caution, as they may also reflect broader processes of professional maturation and workplace socialisation. The findings indicate that introduction programmes that incorporate opportunities for reflection, collegial support, and leadership engagement may help support the continued development of competence and confidence beyond the programme period.

## Background

Societal changes have led to substantial reforms in nursing education, marked by a shift toward increased academization. Baccalaureate education has become the norm and has been associated with improved patient health outcomes [1–3]. However, this academic emphasis has also raised concerns, documented internationally for several decades starting with Patricia Benner in 1982 [4], regarding the balance between theoretical knowledge and clinical skills development. Critics suggest that reduced focus on hands-on clinical training may affect new graduate nurses’ (NGNs) ability to conduct clinical assessments [5–7]. As a result, many NGNs report feelings of insecurity about their professional role and a lack of preparedness for clinical practice. Research from multiple healthcare systems has shown that NGNs often experience difficulties in applying theoretical knowledge to clinical assessments and managing the complex responsibilities of professional practice, including supervision and decision-making [8–10]. These challenges have been linked to discrepancies between academic preparation and the realities of clinical work, making the transition from student to independent nurse a demanding process worldwide [11–14]. Consequently, many NGNs report feelings of insecurity, stress, and uncertainty related to their professional role, contributing to high turnover rates, especially in hospital settings [15, 16].

In response, numerous countries and individual hospitals have implemented introduction programmes for NGNs. In this study, an *introduction programme* is defined as a structured, time-limited initiative supporting newly graduated nurses’ transition into clinical practice through supervised experience and professional guidance. Internationally, comparable initiatives are described using terms such as new graduate residencies, nurse residency programmes, transition-to-practice programmes, preceptorship programmes, and internship models [14, 17–19].

While extensive research has focused on the implementation and outcomes of such programmes, less attention has been given to nurses’ lived experiences beyond the immediate transition period, that is, from a longer perspective and supporting them into their profession. Therefore, this study aimed to explore the lived experiences of being a nurse one year after completing a clinical nursing introduction programme, two years post-graduation.

## Methods

This qualitative study was conducted using semi-structured interviews, with data analysed using thematic analysis based on descriptive phenomenology [20]. Grounded in the work of Husserl [21] and Merleau-Ponty [22], this approach emphasizes the importance of the subjective and lived body in health and care. Phenomenology examines phenomena as experienced by individuals, focusing on the lifeworld perspective, where the body, subject, and world are intertwined [22]. This approach influences data collection and analysis, promoting openness and critical reflection. In line with a descriptive phenomenological orientation and as described by Sundler et al., the analysis focused on identifying and describing themes as patterns of meaning grounded in participants’ lived experiences, while remaining close to the data and without aiming to describe an essential structure of the phenomenon [20]. To report the research, the Consolidated Criteria for Reporting Qualitative Research (COREQ) was used [23].

### The clinical nursing introduction programme

In 2016, the Clinical Nursing Introduction Programme (CNIP) (Table 1) was introduced at a general hospital in southwest Sweden; this programme is still operational. Nurses with a maximum of four months of professional experience are employed within the CNIP. The purpose is to create a safe and supportive environment in which NGNs can progress into the profession through education, clinical supervision, and critical reflection. Distinct from many international transition-to-practice models, the programme integrates formal employment, extended supervised clinical placements across multiple units, structured educational days, and process-oriented nursing supervision with a strong emphasis on critical reflection (Table 1).

**Table 1:** Description of the CNIP’s core components

Core components		Employment and organization	Compulsory introduction week	Placements in different clinical settings	Education days	Process-oriented nursing supervision (POH)
Description	The administrative organization had a unit manager, educational administrator, and programme manager, all full-time. Nurses were employed by the CNIP and worked independently. After completing the CNIP, nurses were offered positions based on their preferences and unit needs.		The introduction week included lectures on the CNIP and hospital employment, practical training, administrative systems, cardiopulmonary resuscitation practice, and a group dynamics seminar.	The CNIP included two placements in different care units. Each placement had a four-week introduction with daily support from a clinical supervisor and mentorship from an experienced nurse. Nurses could also observe at another unit for one week per placement. The placements took place at 6 and 8 months.	During the CNIP, new graduate nurses received 15 days of theoretical and practical education on current topics and were trained using a person-centred approach.	POH is a reflective method in which self-experienced clinical situations are examined from an ethical, patient-centred perspective and in relation to core nursing values. Nurses participate in eight structured group sessions, typically consisting of 6–8 participants, facilitated by a trained supervisor who remains consistent throughout the CNIP. A confidentiality agreement is established at the outset. The group setting is characterised by openness, curiosity, responsiveness, and acceptance, focusing on both patient-related and work-related situations.

### Participants

Participants were recruited using convenience sampling [24] and consisted of 12 registered nurses (RNs) who were two years post-graduation from nursing school and one year after completing a clinical nursing introduction programme (CNIP). Among the 119 nurses who completed the CNIP, all were invited to participate in individual interviews one year after programme completion (2019–2020 cohorts), of whom 12 agreed to take part, corresponding to a participation rate of 10.1%. Participant characteristics are presented in Table 2.

**Table 2:** Characteristics of the participants

	*N* = 12
Age	24–48 year
Sex	2 males and 10 females
Placements in the CNIP that was performed	One placement: *n* = 6 (due to the pandemic)
	Two placements: *n* = 6
Change of workplace and employment immediately after the CNIP	Remaining at one of the placements: *n* = 5
	Another ward within the hospital: *n* = 1
	Municipal care: *n* = 3
Undergoing or finished postgraduate studies	Remaining at one of the placements plus specialist nursing postgraduate programme: *n* = 2 (elderly care, psychiatry)
	New ward at the hospital including specialist nursing postgraduate programme: *n* = 1 (intensive care)

### Data collection

Individual semi-structured interviews were conducted from 2021 to 2023 to capture participants’ experiences. Written and verbal information about the study was provided at the start of the CNIP, and written information was sent by post for the current study. Interested participants were contacted by researchers, and informed consent was obtained. A minority of participants also participated in interviews at programme completion reported elsewhere [25]. Participants chose the time and place for the interviews, with all opting for digital interviews with a visual option (*n* = 12). The interviewers were not involved in the organization, delivery, or assessment of the CNIP and held no managerial or evaluative roles in relation to the participants, thereby minimising potential power imbalances. A third researcher was involved in the organization of the CNIP but did not conduct interviews to avoid influencing the interview process; instead, she entered the study at a later stage to minimize bias and contributed programme-specific expertise, playing a critical role in refining and validating the data analysis conducted by the other authors. Prior to data collection, participants were informed of the researchers’ roles and the voluntary nature of participation to support openness and reduce the risk of socially desirable responses.

A semi-structured interview guide with open-ended questions was used (Table 3), starting with the instruction, ‘Please describe how it feels for you now to be a nurse with two years’ work experience?’ Follow-up questions, such as ‘How have you developed during this time?’ and ‘What are your thoughts on the CNIP a year after you completed it?’ were added as appropriate. Clarifying questions such as ‘What do you mean by that?’ encouraged participants to elaborate, providing a richer view of their experiences and helping interviewers confirm their understanding. The interviews lasted between 28 and 51 min (mean = 36) and were digitally recorded. No field notes were taken.

**Table 3:** Research interview guide

Focus Area	Interview Questions
Current professional experience	Can you describe how it feels for you right now to be a nurse with two years of professional experience?
Professional development	In what ways do you feel you have developed during this time?
Sense of security in the professional role	How do you experience a sense of confidence or security in your professional role, and how has this changed over time?
Challenges	What has been particularly challenging during this period?
Support for development	What has particularly supported your development?
Reflections on the CNIP (one year later)	How do you think about the CNIP now, one year after completing it?
	• What worked well?
	• What could have worked better?

### Data analysis

In this study, a qualitative thematic analysis grounded in descriptive phenomenology was used to explore nurses’ lived experiences from a lifeworld perspective [20]. The analysis was guided by a phenomenological attitude, emphasizing openness to participants’ experiences and bracketing preunderstandings. All interviews were transcribed verbatim and analysed iteratively, moving back and forth between the whole and parts of the text to capture the essential meanings of the phenomenon. Inspired by Sundler et al., the analysis involved identifying meaning units that were closely related to the study aim. These meaning units were compared for similarities and differences, condensed, and abstracted into codes. The codes were then grouped and clustered into patterns of meaning through an inductive and iterative process. Themes were developed to capture both variations and commonalities in participants’ lived experiences, following the analytic steps outlined in Table 4.

Throughout the analytic process, preliminary meanings and patterns were noted and continuously discussed among the authors. Visual strategies, such as colour-coding and mapping of relationships between codes, were used to facilitate the organisation and refinement of emerging themes. Themes were revisited, compared, and where appropriate merged, ensuring that they remained grounded in the data and in line with the study aim.

To support bracketing, the primary analysts engaged in continuous reflection on and articulation of their preunderstandings throughout the analytic process. Additionally, a third researcher, who had not been involved in the interviews, was introduced at a later stage to provide an independent and critical perspective. This contributed to challenging underlying assumptions, reducing the risk of interpretive drift, and strengthening analytic rigour. Throughout the analysis, preunderstandings were actively reflected upon and deliberately restrained to avoid premature interpretations and to maintain sensitivity to emerging meanings.

**Table 4:** Overview of the steps of the analysis process

Step	Thematic analysis
I	**Open-minded repeated** *reading of the transcripts to gain an overall understanding and remain open to new and unique meanings related to the phenomenon of being a nurse one year after a clinical nursing introduction programme*,* two years post-graduation.*
II	The text was reread with a focus on identifying **meaning units** that reflected participants’ lived experiences of the phenomenon. These meaning units were highlighted and briefly described to stay close to the original data.
III	Meaning units were compared for similarities and differences **and systematically grouped into patterns**. Through a reflective process, condensed descriptions were developed into **themes** expressing the essence of the phenomenon. Quotations were used to illustrate how the themes were grounded in the data.

## Results

The analysis resulted in three themes, *a hunger for more*, *responsibility for being a caring nurse*, and *becoming self-aware and self-confident*. The results are presented in the following subsections and illustrated by quotations from the respondents.

### A hunger for more

Two years after graduation and one year following participation in a clinical nursing introduction programme meant a willpower and strong strive for ongoing progression in the profession and as a person, still aware that there is much more to learn:

You are definitely not complete.

The professional hunger appeared as an interest and a curiosity. It was also expressed as a will to take responsibility and to be more independent at work with the goal to work autonomously and constantly seek more and deeper knowledge.

Partaking in the CNIP was described as an opportunity to work in different units and wards at the hospital during the first year as a nurse, where the CNIP laid the foundation to make it possible to become a nurse with different skills and competencies. For some of the nurses, the change of workplace to a different ward led to progress and stimulus, while others wished to stay and deepen their knowledge at the first workplace. The hunger for more was also expressed in a wish to combine work in hospital with working in primary health or municipal care within the CNIP and continue working as a nurse to make constant professional progress.

The nurses either took great responsibility for the progression of nursing care in their workplaces, or they undertook education to specialize within their profession, for example by enrolling in a master’s programme. Sometimes they combined the work with education to deepen their knowledge:

I mainly work alone, and I feel that it is comforting to have more support within myself with deepening my knowledge.

A hunger for more was also shown in the willingness to be able to combine work and further studies with private life, such as starting a family. If their hunger was not satisfied at their current unit, they moved on and changed workplace.

The hunger for knowledge made participants impatient, and they took the responsibility for their learning seriously:

I think it takes too long before you start to find your feet, before you’re fully trained, and everything starts to make sense. I felt that I needed—and wanted—to be able to go to work as quickly as possible and feel calm and secure, no matter what kind of patients I was going to meet. I felt that I needed to read books and search online for information.

The hunger for more was driven by personal and professional visions and goals; as soon as a goal was achieved, another one was set. This applied to both clinical work as nursing care for patients and their personal growth and was performed both individually and in teams with colleagues:

You plan together with the team, and then the team works towards achieving the goals you’ve set. So, I think I’ll stay and work in this unit to try and learn that way of working.

A negative aspect of always having a hunger for more is that it costs energy. The hunger or the continuous hunt for more knowledge was experienced as aging quickly while transitioning into the profession. Many felt drained and fatigued. Although it was being perceived as positive to have this hunger, it came at a cost. Despite the ambition to learn more and to do more, participants also expressed the desire to be humble:

There is a desire to remain humble, recognizing that learning is a continuous process and that new insights are always possible.

### Responsibility for being a caring nurse

One year after participating in the CNIP, the responsibility for patients’ care was at the forefront for participants. A willingness to provide a caring environment was essential, to the extent that nurses would rather leave their workplace than support an uncaring organization. Caring is at the foundation of being a nurse and something to be proud of, as participants wanted to use their caring competence in their daily work. They understood that caring is not entirely up to themselves but could be affected by the structure of the workplace that is failing:

I’ve done the best I can but still don’t have time to provide the care that patients are entitled to. Then it’s not just on me—it’s on the organization.

The responsibility for being a caring nurse is highly regarded, and participants were not willing to forsake a caring attitude. To be a caring nurse was vital as it was at the core of their profession and themselves as persons. The independence of the nursing profession and the professional caregiver was what it was all about. There was a strong desire and ambition to see the patient as a person and not as an object. Giving the patient the care they need and are entitled to from the ethical and professional points of view was crucial:

What tools can we offer to improve their life situation in the long term and follow how they develop—that’s something I really enjoy. I guess I missed that when I worked in orthopaedics, where it was mostly in and out, in and out, getting patients home as quickly as possible.

When the organization did not enable them to be true to themselves and when caring was lost, this was expressed as the nurse being lost. Participants identified with being a nurse, their professional role as a caring person, to such an extent that nursing was closely intertwined with their sense of self. Consequently, situations in which they were unable to practise nursing and caring as they believed they should were experienced as a personal loss. Feelings of sadness and despair arose when participants were unable to provide good care and was perceived as a failure to uphold the core values of their profession. Maintaining professional pride and not compromising on who they were was a priority for participants. The desire to meet patients’ needs, provide care, and engage in conversation was fundamental. This commitment reflects a deep sense of professional pride and dedication to the well-being of others:

I find it stimulating and it feels good in my soul when I feel that I’m doing a good job and helping people.

Providing good care provides nurses with much in return, and many meaningful relationships were created because many of the patients were very lonely. Deep caring relationships were important for the nurses, as they are at the foundation of caring:

It gives so much back when you feel that you’ve reached someone and see that it leads to a major life change. When you actually get beneath the surface and connect with them on the inside. The situation is serious, and we need you to get on board—not just say yes and then go home and do something else.

Part of the challenge was working more independently, as being a nurse is a highly autonomous role. Another challenge, and often really appreciated—was the opportunity to build ongoing patient relationships, which risked being missed due to the heavy workload of the ward. If the organization was not supporting caring, some of the nurses changed workplace, sometimes within the hospital:

One of the reasons I applied to work in the outpatient setting was the opportunity to care for long-term patients—many of whom remain with us for up to ten years. This continuity allows for the development of meaningful care relationships, which I especially value.

An additional dimension in the development of being a caring nurse was the role of life maturity, which contributed to a deeper capacity for empathy, reflection, and relational understanding. When nurses experienced a sense of groundedness, personal security, and maturity, this was experienced as being reflected in the quality of care they provide. Such inner stability facilitates the capacity to go beyond routine tasks, enabling the initiation of difficult conversations—particularly those concerning existential matters—with greater openness and sensitivity:

I remember when I had just graduated as a nurse, I felt like—well, we can’t just sit there and tell people they’re going to die, that’s not possible unless they’re actually dying. But today, I feel that I can do that. I can really make it clear that things are that serious.

### Becoming self-aware and self-confident

One year after participating in the CNIP, participants described experiencing an increased feeling of security in the profession. This security was also felt personally:

I feel that I’ve developed a lot as a person and become much more confident in myself. Since I work so independently, I can see the results, and I think the development of that confidence has been very strong.

Asking for help was no longer considered a weakness rather a sign of being secure among colleagues, as an expression of being more confident and secure in the profession. Becoming more self-aware in the nursing profession was related to the nurse as a person. The development of this feeling of security sprung from the support of colleagues but also from caring relationships with patients. Peers and colleagues offered support when they reflected on ethical dilemmas together. The CNIP contained reflective supervision in groups. These reflective supervisions are missing today, but sometimes reflective moments are offered by colleagues:

It’s the opportunity to discuss and reflect, to step outside your formal role and just be a nurse—sharing what happened, what we did, and asking: what would you have done? I really value that group reflection, finding different solutions to the same situation, because my solution isn’t necessarily the best one.

The reflective attitude contributes to becoming more self-aware. The CNIP was experienced as giving a solid foundation for becoming a confident nurse. The programme, which contained lectures, reflective supervision in groups, and being placed in different clinical settings, led to a preparedness for clinical work as a nurse:

What I experienced as very positive was that I felt well prepared when I started my permanent position. After completing the CNIP, I had already done all the basic training—I was ready. That meant I could focus fully on starting the actual work, and that felt really, really good.

Experiences from various workplaces provided valuable insights into both individual and team dynamics, which in turn contributed to an increased sense of security in the professional role. The experience of encountering varying expectations from colleagues unfolded gradually; at first, there was a sense of being considered newcomers navigating unfamiliar routines, but over time, as they became more attuned to the workplace, colleagues’ expectations shifted—reflecting a deepening sense of belonging and professional identity. Simultaneously, as life itself evolves and new experiences emerged, a deeper sense of security was established. Through an ongoing awareness of one’s own capabilities and limitations—recognizing areas of strength as well as those that present challenges—a more grounded understanding of the professional self and its relational context began to take shape.

Not everything was supportive and well-functioning during participants’ initial time as nurses. The CNIP comprised several parts, and the part in the different wards and units sometimes lacked structure in terms of introduction and supervision:

I didn’t get proper introduction and initial mentoring because there simply wasn’t time for it, and things were constantly chaotic. That was really unfortunate, because I actually think the CNIP is a great concept—when it works well.

Working as a nurse also meant making many decisions, which was experienced as difficult when participants were new in the profession. Being more secure as a person meant that it was easier to manage challenges at work. Being new was experienced as entailing fewer demands, which was comforting. There was an acceptance from the manager and colleagues that they were new and were allowed to be new, that they did not have to know everything. The elements within the CNIP that had helped them was the chance to get away from the ward, to go to lectures, and to meet peers. They could have reflective discussions with the other new nurses, which felt reassuring; they were not alone in having these feelings of not knowing everything and that was okay. The management of the CNIP was another element that was experienced as being helpful if something needed to be addressed in a more formal manner:

Overall, I found the experience to be positive. I appreciated the educational sessions and the opportunities to engage in reflective conversations during group meetings. It was also reassuring to have a manager to turn to, especially when I felt the need to step away from my unit—perhaps due to not feeling comfortable or facing challenges in collegial relationships. In such moments, having another point of connection or belonging offered a sense of relief and support.

The CNIP became a respite from everyday work while still enabling participants to acquire knowledge without the stress from the ward.

## Discussion

This study aimed to explore the lived experiences of being a nurse one year after completing a clinical nursing introduction programme, two years post-graduation. The findings suggest that CNIP may have been experienced as a contributing factor for both professional and personal growth as nurses became more established in their roles. The findings elicited the following themes: *A hunger for more*,* responsibility for being a caring nurse*,* becoming self-aware and self-confident*. The essential CNIP components perceived by participants as valuable for their development included time away from clinical duties, peer interaction, reflective dialogue, and supportive management. Similar findings from an introduction programme were reported by Lindblom et al. [26]. Specific to the present study is that these components together with broader professional and personal experiences, were described as continuing to influence nurses’ ongoing professional development beyond the CNIP period.

Previous research has demonstrated the benefits of structured nurse residency programmes in terms of competence, retention, and job satisfaction [27, 28]. By contrast, the present study does not seek to evaluate the outcomes of the CNIP, but to deepen understanding of how nurses experience and interpret their professional development over time. By focusing on nurses’ reflections one year after completing the CNIP, this study contributes insight into how early support may be integrated into clinical practice over time, an aspect that has received less attention in existing research.

The nurses expressed a hunger for more and wanted to continue their learning through education, a routine that they became accustomed to during the year they participated in the CNIP. The nurses were used to having regular lectures and this may have influenced them in wanting to know more. Mlambo et al. [29] found that a supportive environment is a prerequisite for continuous professional development (CPD), and similar findings were reported in a scoping review [30]. This may represent an advantage of CNIP and similar introduction programmes, as they may help to facilitate a foundation for CPD. The sustained motivation observed among participants was described in relation to the process-oriented group supervision (Table 2), where structured reflection and facilitated dialogue support both coping and professional growth. Such mechanisms have previously been associated with reduced stress and the maintenance of learning over time [31, 32]. Nevertheless, it cannot be determined to what extent these experiences are attributable specifically to the CNIP, as opposed to ongoing clinical exposure and development within the workplace context.

The transition from ‘being’ to ‘becoming’ a nurse reflects a dynamic and ongoing process of professional identity formation. It is not a fixed state, but a continuous journey shaped by experience, reflection, and evolving competence—highlighting nursing as a lifelong learning profession. *Being*, moving from a beginner to someone who can look beyond their abilities, or inabilities, to see the patient, has been described in line with the essence of Duchscher’s [33] and Benner’s [4] models. Murray, Sundin, and Cope [34] emphasise that NGNs evolve through Benner’s ‘novice-to-expert’ stages and Duchscher’s transition shock framework, enabling them to transcend technical uncertainty and focus holistically on patient care. Benner [4] describes this evolution as a shift from reliance on abstract principles to using concrete experiential paradigms, marking progress toward proficient, intuitive practice. Ultimately, nurses’ ability to look beyond personal limitations signifies a deepening clinical insight and reinforces lifelong learning within professional development.

*Becoming*, on the other hand, represents an active, forward-looking process of growth—embracing curiosity, seeking knowledge, and striving for autonomy while integrating caring values into practice [35]. It refers to a state of continuous progression where professional confidence and ethical awareness mature alongside personal development. This duality of being and becoming emphasizes that nursing identity is never static; it evolves through reflection, supportive environments, and meaningful patient relationships, forming the foundation for sustainable and resilient practice. In this context, the CNIP may be understood as one of several contributing influences rather than a singular determining factor.

Nurses’ experiences of being in the profession one year after a clinical introduction programme and two years post-graduation reveal a persistent drive for growth. The question arises: Does this continuous striving risk contributing to stress? Research shows that early‑career nurses often feel pressure to develop and adapt, which can lead to feelings of being overwhelmed [36]. At the same time, such characteristics are fundamental to lifelong learning—a process that enhances clinical judgment, care quality, and patient outcomes [36]. This is associated with the concept of ‘becoming’, which promotes well‑being in nursing [37]. Furthermore, the theme of responsibility for being a caring nurse reflects a progression toward self‑awareness and self‑confidence. These qualities may serve as protective factors against the negative effects of stress [38]. A central question remains: Is it acceptable to pause and simply be a competent nurse—feeling satisfied with oneself? Allowing such moments of contentment may represent professional maturity and a strategy for sustainable practice. Future research incorporating longitudinal designs, comparison groups, or triangulated data sources would be valuable in further clarifying the specific contribution of introduction programmes such as the CNIP.

### Strengths and limitations

A key strength of this study is its follow-up design, which captures nurses’ reflections one year after completing the CNIP. While research on newly graduated nurses often focuses on experiences during or immediately following programme participation, this study extends previous work [25] by exploring how nurses, after an additional year of professional practice, interpret their earlier experiences and perceive the programme’s relevance to their ongoing clinical work. The phenomenological approach enabled an in-depth exploration of lived experiences, and methodological rigour was enhanced through researcher triangulation, reflexivity, and the use of established quality frameworks, including the COREQ checklist [23] and Lincoln and Guba’s criteria for trustworthiness [39].

The findings should be interpreted considering the study context. Data collection concerned nurses who participated in the CNIP during the COVID-19 pandemic, a period marked by disruptions to both healthcare delivery and nursing education. Reduced training and supervision opportunities may have influenced participants’ experiences of preparedness, support, and professional development [40]. In addition, variation in programme exposure resulting from pandemic-related adaptations may have affected participants’ experiences, although exploring such differences was beyond the scope of this study. These contextual factors may nevertheless have influenced participants’ experiences and should therefore be considered a limitation.

Several methodological limitations should also be acknowledged. The use of convenience sampling and recruitment from a single hospital may limit the transferability of the findings. Self-selection may have influenced participation, and because no formal sampling frame or recruitment rate was recorded, the extent of potential selection bias cannot be assessed. The use of digital interviews may also have affected the depth of the data by limiting access to non-verbal and contextual cues, although some participants may have felt more comfortable sharing their experiences in this format. Furthermore, the absence of field notes limited opportunities to contextualise and enrich the interview data.

Although the findings may be relevant to similar settings where structured introduction programmes are implemented, caution is warranted in interpreting them as evidence of programme effects. The study relied on retrospective self-reported experiences and did not include comparison groups, pre–post measures, or additional data sources. Participants’ accounts may therefore also reflect broader processes of professional maturation and workplace socialisation. Furthermore, data were collected at a single time point, limiting insight into how experiences and perceptions may change over time. Longitudinal studies with multiple follow-up points would provide a more comprehensive understanding of nurses’ development during the early career period.

Despite these limitations, this study contributes valuable insight into the early transition phase of nursing practice. The transition from novice to practicing nurse is widely recognised as a demanding and complex period. While previous research highlights the potential benefits of structured introduction programmes, longitudinal perspectives remain scarce. This study therefore addresses an important gap by providing a deeper understanding of how nurses reflect on and interpret their early professional development beyond the immediate programme period.

## Conclusions

The findings suggest that participants perceived the Clinical Nursing Introduction Programme as providing valuable support for their ongoing professional and personal development. Participants highlighted protected learning time, structured reflection, peer support, and accessible leadership as important aspects of the programme. These elements were described as facilitating the transition from ‘being’ a novice nurse to becoming’ a confident and reflective practitioner. These elements were perceived as supporting the development of clinical judgement, reinforcing caring values, and encouraging engagement in lifelong learning.

Participants also perceived that the programme’s influence extended beyond its formal duration, contributing to their sense of wellbeing, resilience, and commitment to person-centred care. While these findings are limited to the experiences of nurses within the study context, they indicate that introduction programmes incorporating opportunities for reflection, collegial support, and leadership engagement may help support nurses during the early stages of their careers. Further research is needed to explore the longer-term effects of such programmes on professional development, workforce retention, and patient care.

## Data Availability

The data underlying this study may be obtained from the corresponding author on reasonable request.

## Abbreviations

CNIP: Clinical nursing introduction programme
CPD: Continuous professional development
NGNs: New graduate nurses
RNs: Registered nurses
